# Breast Augmentation by Fat Transplantation With Adipose-Derived Stem/Stromal Cells

**DOI:** 10.1093/asjof/ojaa007

**Published:** 2020-02-07

**Authors:** Dong-jin Shin

## Abstract

**Background:**

Surgical methods using implants were broadly selected for breast augmentation surgery until recently; however, owing to several associated problems, fat transplantation using adipose-derived stem/stromal cells (ADSCs) has been suggested as an alternative.

**Objectives:**

This study evaluated the clinical benefits of fat transplantation using ADSCs for breast augmentation.

**Methods:**

The clinical effects were investigated in 105 patients who underwent breast augmentation with ADSCs and fat transplantation. Liposuction was performed in the abdominal and/or thigh regions; ADSCs were isolated from the fat, mixed with refined fat, and transplanted into each breast; and changes in the breast volume were measured.

**Results:**

The average increase in breast volume was approximately 185 mL at 2 weeks after operation. Fat engraftment rates were 85.1, 75.1, and 73.7% of augmented volumes after 1, 3, and 6 months, respectively. A total of 39 patients who received >60 million ADSCs exhibited a transplanted fat engraftment rate of 90.5% (average increase, 162 mL), whereas this rate was 68.9% (average increase, 115 mL) in 31 patients who received <60 million ADSCs.

**Conclusions:**

This study demonstrates that breast augmentation with ADSCs and fat transplantation is effective. Surgical outcomes substantially improved with increased numbers of implanted ADSCs.

**Level of Evidence: 4:**

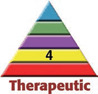

Fat transplantation is a method used to augment the pre-existing anatomical volume by obtaining the patient’s fat, refining it, and then injecting it in the desired region. This surgical procedure is widely practiced because procuring fat tissue is relatively easy and transplanted tissues frequently result in a natural outcome with no immunological rejection or foreign body reaction. However, the associated disadvantage is the low-fat engraftment rate after surgery (most of the fat is absorbed). Substantial fat transplantation is required for adequately augmented breasts; however, complications of fat necrosis or calcification are often observed, thus decreasing the engraftment rate.

Zuk et al^1^ successfully derived stem cells from human adipose tissue and, subsequently, several studies on adipose-derived stem cells (ADSCs) have been reported. Furthermore, although fat transplantation with ADSCs has become more prevalent, statistically significant comparative findings remain lacking and the true efficacy of fat transplantation with ADSCs remains controversial.

Breast augmentation with fat transplantation and ADSCs has been performed at SC301 Cosmetic Clinic, Seoul, South Korea, since 2007. The outcomes and results of breast augmentation obtained in 2012 or later have been subjected to statistical analyses. This prospective study collected information on the long-term clinical outcomes of 105 patients who underwent stem cell-based breast augmentation at the clinic from January 2016 to July 2016.

## METHODS

### General Data

This study evaluated the information of healthy women aged 21 to 55 years with no pre-existing history of breast augmentation with ADSCs. All patients included in the study visited the SC301 Cosmetic Clinic. In total, 105 patients who received 150 to 314 mL of fat through chest injections, including those who received breast implants, were analyzed. Thirty-five patients with irregular or omitted measurement values because of failed postoperative follow-ups were excluded.

The study was conducted in accordance with the Declaration of Helsinki. The research objective; surgical methods; and associated caution, risks, and side effects were thoroughly explained to the patients, and written consents were obtained from them before surgery.

### Surgical Procedures

#### Preoperative Management of Patients

Each breast of all the patients, excluding those with sagging breasts or those who had their implants removed, was injected with 50 mL normal saline or PDRN (polydeoxyribonucleotide sodium), and the breast space was expanded using equipment (HRME, Seoul, Korea) that applies negative pressure to each breast to increase the breast volume before surgery. This procedure was completed in 1 h and was repeated five times in every patient. In specific patients, such as those with dense breasts, the intensity and frequency of care was increased depending on the degree of their condition.

Elasticity of the breast was evaluated preoperatively in the surgical room using the pinch test on the breast skin. The injection volume was constant despite the varying elasticity. Two patients removed their implants, and partial capsulectomy was performed at the time of removal.

### Fat Collection

In general, 2000 mL of tumescent solution with a local anesthetic solution was evenly injected into each desired region for fat extraction, while the patient was under sedation/anesthesia. The tumescent solution comprised 2 L of normal saline, 40 mL of 2% lidocaine, and 2 mL of epinephrine. The fat was then extracted using a cannula (3 mm × 30 cm) suction pipe at a pressure ≤500 mmHg (Supplementary Figure 1).

### ADSC Isolation

Impurities were removed from the extracted fat by centrifugation followed by pipetting. ADSCs were isolated from 60 mL of purified fat using the Icellator cell isolation system (Tissue Genesis, Honolulu, HI, USA) (Supplementary Figure 2). In case of excess fat, 60 mL of fat was manually obtained using the cell extraction method, and 15 to 20 mL of isolated cells were diluted for subsequent use.

### Fat Transplantation

Before injection, a small hole was made on the lateral superior part of the breast, and a 2-mm diameter cannula (20 cm long) was used to transplant the refined fat into the breast; 9 mL of refined fat and 0.5 mL of diluted ADSC suspension were mixed to make 15 to 20 mL of ADSC dilute solution. The combined tissue was then injected into the lower region of the pectoralis major (50%) and the region above the pectoralis major (25%); the remaining 25% was injected into the breast tissue and subcutaneous tissue layers (Supplementary Figure 3). If no complications were noted, the remaining fat mixture was completely injected. In total, 3 × 10^7^ to 2.6 × 10^8^ ADSCs were used per patient, and a total volume of 150 to 314 mL was injected into on each breast.

### Postoperative Management of Patients

The patients were advised to visit the clinic approximately 3 months after surgery when engraftment of the transplanted fat was approximately complete. During the initial 3 months, the patients were advised to refrain from smoking, perform strenuous exercises involving the arms, and use sauna. A high-protein diet was recommended, and reduced intake of food was prohibited. Body fat percentage was recorded at each visit to measure any decrease in weight or body fat percentage.

### Evaluation

The breast volume was measured and analyzed with Axisthree^®^ (Axisthree, Germany) preoperatively and 1 to 2 weeks, 1 month, 3 month, and 6 months postoperatively (Supplementary Figure 4). Cell counts were measured with ADAM (NanoEnTek, Korea). The results were compared based on valid cell counts of <60 million and >60 million nucleated cells (Supplementary Figure 5). Elasticity of the breast was examined during surgery and at postoperative visits.

Information of patients who were injected with <150 mL of fat graft and those who did not visit the clinic postoperatively was not included in statistical analyses. The initial augmented breast volume was measured 1 to 2 weeks postoperatively after the swelling had subsided.

## RESULTS

### General Status

The surgery was completed in all the 105 patients (cases 1–105), and no unexpected complications or specific adverse effects were observed. The mean age of the patients was 34 years (range, 21–55 years). The patients were advised to visit the clinic for 6 months postoperatively. They were also notified of other cautions, such as refraining from using their arms for 3 months, and were restricted from severe weight control. Remarkably, in a satisfaction survey at the end of the 3-m follow-up, most patients reported a soft and natural feeling after surgery and expressed contentment with their augmented breasts.

However, the author received this opinion on the metrics of this procedure during postoperative interviews with the patient but did not obtain numerical or detailed statistical data.

There was no statistical significance owing to several reasons. First, the assessment was not conducted anonymously. Second, the evaluators were frequently changed. Finally, there were several missing records. In cases with differing left and right injection volumes, the breast injected with a higher volume was evaluated, whereas in cases with equal injection volumes, the left breast was evaluated to maintain consistency. The patients were followed up from 2 weeks to 6 months postoperatively, and the mean follow-up was 1.84 months.

### Analysis of Clinical Effects by Period

**Figure 1.**
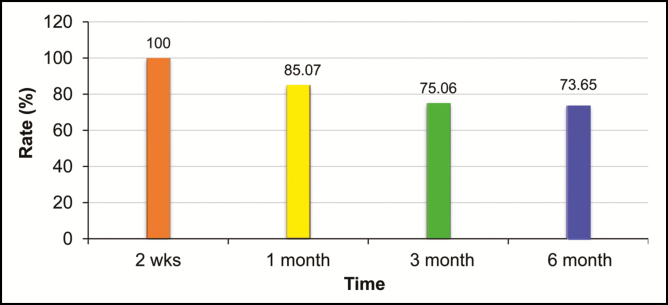
Comparison of augmentation effects after breast augmentation with ADSCs (adipose-derived stem/stromal cells) and fat transplantation

Before surgery, the total volume of the breasts of 105 patients was 20,778 mL, and the mean volume was 197.89 mL (range, 82–432 mL). The total increase in the patients’ breast volumes within 1 to 2 weeks postoperatively was 19,379 mL, and the mean volume was 184.56 mL (range, 122–352 mL). Within 1 month postoperatively, the total increase in the breast volumes of 86 patients who visited for follow-ups was 13,725 mL, the mean volume was 159.59 mL (range, 94–314 mL), and the retention rate was 85.07%. Within 3 months postoperatively, the total increase in the breast volumes of 70 patients who visited for follow-ups was 9753 mL, the mean volume was 139.33 mL (range, 75–300 mL), and the retention rate was 75.06%. Within 6 months postoperatively, the total increase in the breast volumes of 32 patients who visited for follow-ups was 5056 mL, the mean volume was 158.00 mL (range, 83–277 mL), and the retention rate was 73.65% (Figure 1).

### Analyses of Clinical Outcomes Based on the Number of ADSCs Transplanted

The valid cell counts were measured (range, 30–206 million nucleated cells per 60 mL lipo-aspirated fat tissue), and among 70 patients who visited the clinic 3 months postoperatively, the fat engraftment rate in 31 patients who received 30 to 60 million ADSCs was 69%; the average increase in the breast volumes was 115 mL. The fat engraftment rate in 39 patients who received 60 to 206 million ADSCs was 80%, and the average increase in the breast volume was 162 mL.

### Effect of Breast Skin Elasticity on Clinical Outcomes

**Figure 2.**
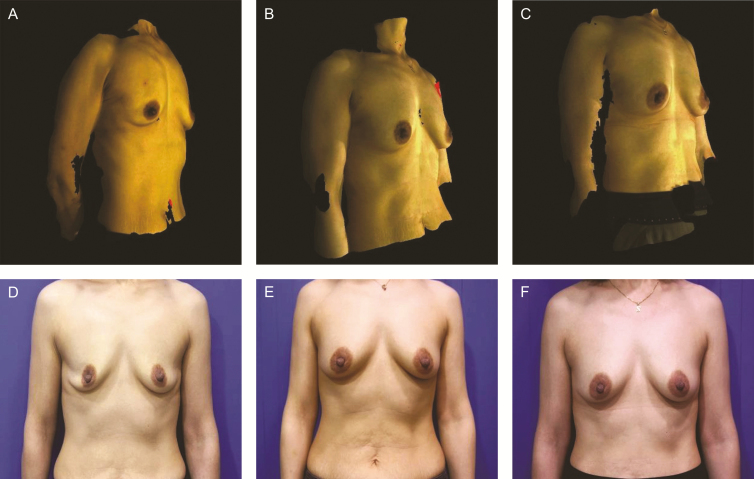
Case 1, a 44-year-old female patient with sagging breasts after breastfeeding and small breasts. (A, D) Preoperative images, breast volumes of 140 mL (right) and 168 mL (left). (B, E) Three-month postoperative images, breast volumes of 284 mL (right) and Lt 280 mL (left). (C, F) Six-month postoperative images, breast volumes of 230 mL (right) and 269 mL (left).

**Figure 3.**
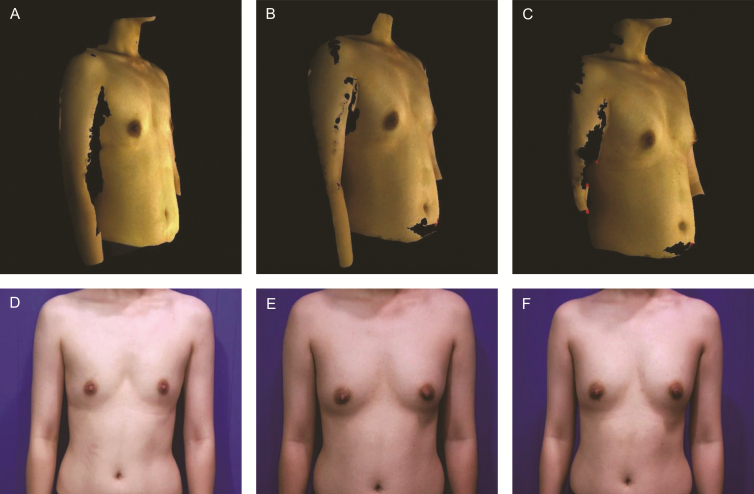
Case 2, a 24-year-old female patient with small breasts. (A, D) Preoperative images, breast volumes of 139 mL (right) and 151 mL (left). (B, E) Three-month postoperative images, breast volumes of 232 mL (right) and Lt 238 mL (left). (C, F) Six-month postoperative images, breast volumes of 237 mL (right) and 240 mL (left).

Among the 70 patients who visited the clinic at 3 months postoperatively, elasticity of the breast skin was classified as “fair” for 37 patients and “relatively insufficient” for 33 patients. For patients with fair elasticity, 81% of the breast volume was maintained with an increase of 165 mL from baseline. For example, the elasticity was classified as fair for case 1; the breast volume of case 1 was Rt 325, Lt 353 mL at 2 weeks postoperatively. Therefore, in the right breast, this patient was maintained with an increase of 144 mL and 78% of the breast volume within 3 months postoperatively (Figure 2). For patients with relatively insufficient elasticity, fat engraftment rate was 68% with an average increase of 113 mL in breast volume. For example, the elasticity was classified as relatively insufficient for case 2; the breast volume was Rt 278, Lt 345 mL at 2 weeks postoperatively. Therefore, in the right breast, the patient was maintained with an increase of 93 mL and 67% of the breast volume within 3 months postoperatively (Figure 3). Although the statistical analysis of skin elasticity relative to the number of ADSCs was required, it was not performed because the number of patients was considered very small for further divisions. Furthermore, we believed that this would result in low statistical reliability.

## DISCUSSION

Fat transplantation that was first applied >100 years ago in the field of cosmetic and aesthetic surgery has proved to have important advantages.^2^ Despite these advantages, a lack of fat engraftment after surgery has been a persistent issue and long been researched with profound interest by several investigators. Since the discovery of ADSCs, fat transplantation has received more interest from cosmetic surgeons with a particular interest in ADSC functions. A previous study reported that ADSCs can differentiate into adipocytes, osteocytes, chondrocytes, and neurocytes.^3^ Adipose-derived stem/stromal cells are also capable of secreting various growth factors, including vascular endothelial cell growth factor, hepatocyte growth factor (HGF), and insulin-like growth factor-I (IGF-1).^4^

Matsumoto et al^5^ originally reported the concept of fat transplantation with ADSCs and demonstrated that cell-assisted lipotransfer (CAL) could result in an increase of approximately 35% in engraftment rates, which is higher than the engraftment rate for simple fat transplantation. In principle, fat from any part of the body can be collected for fat transplantation. Additionally, the engraftment rate of subcutaneous fat, which may be collected from the abdomen and thigh regions, is higher than that of visceral fat.^6^ Another study has reported that the survival rate of adipocytes in transplanted fat increases in the presence of isolated ADSCs.^7^ In a comparative study on ADSC levels within surgically excised adipose tissue and aspirated adipose tissue, Matsumoto et al^5^ reported that ADSC levels in aspirated adipose tissue were only 50% of those in excised adipose tissue. There were two reasons for this finding: (1) most ADSCs were located proximally to large blood vessels and remained there even after liposuction and (2) during liposuction and cell isolation, ADSCs present in the adipose tissue were damaged, thus losing their intrinsic function. Thus, Matsumoto et al^5^ concluded that the effects of fat transplantation were insufficient because of the impaired ADSC function after liposuction and isolation. Recent technological innovation has minimized this type of damage, and improved equipment now allows the isolation of large quantities of ADSCs.

Several preclinical and clinical studies have reported that a mixture of ADSCs can increase the survival rate of adipose tissue after fat transplantation^8^ through the following mechanisms: (1) ADSCs can facilitate regeneration of adipose tissue by differentiating into adipocytes; (2) they can induce vasculogenesis within adipose tissue by differentiating into vascular endothelial cells and vascular wall cells; (3) they can promote remodeling of the environment around the adipose cell and form blood vessels by secreting growth factors such as VEGH, HGF, and IGF-1 within the hypoxic environment; and (4) their contents are continuously maintained by persistent division of existing ADSCs.

After confirming that ADSCs increased the engraftment rate of transplanted fat in 2007, the author has continued to perform breast augmentation with ADSCs and fat transplantation. The present study excluded patients with fat injection of <150 mL to increase the precision of volume measurements. Patients who sporadically appeared for follow-up were also excluded. The left and right injection volumes differed in several patients, and the standard was preferentially set to the side with greater injection volume.

Although the same volume of tissue was transplanted into each breast, the volume of expansion often differed between the two breasts possibly because of patients’ lifestyles after surgery. For instance, repeated use of the arms decreases the survival rate of fat tissue transplanted to the breast. The engraftment rate decreased in the breast on the same side as the arm that was frequently used postoperatively. We were unable to quantify the use of the arms to demonstrate that the degree of arm use was important for survival of the grafts.

Patients with the following conditions also relatively exhibited poor clinical outcomes after surgery: (1) Patients with relatively thick skin of low elasticity often had insufficient space for breast augmentation. Thus, even when a relatively large volume of adipose tissue was transplanted, breast augmentation remained indistinct within the predicted period. For these patients, the clinical outcomes could be improved through premanagement at the clinic. (2) A decreased survival rate of fat tissue was observed in patients who exercised, which requires frequent movement of pectoralis. In such patients, a large volume of adipose tissue was damaged or absorbed after surgery. Thus, patients desiring optimal clinical outcomes should follow postoperative management for proper guidance on appropriate exercises. Although the left and right breasts were similar, the right breast was typically injected with an additional 20 mL of fat. (3) In patients with restricted diets after surgery, engraftment rates may have decreased because of a lack of nutrition to the transplanted fat and ADSCs. Thus, clinical outcomes may be improved by recommending regular diet with extra protein for 3 months postoperatively.

Fat transplantation with CAL is a transplantation surgery performed with ADSCs. Although it involves less possibility of complications compared with conventional fat transplantation, excessive fat transplantation and decreased engraftment remain to be associated with postoperative complications, such as fat necrosis, calcification, multiple cystic lumps, and pseudotumor.^9^ However, in this study, the adverse effects were fewer with breast augmentation using fat transplantation with ADSCs than with the conventional fat transplantation method. Moreover, the symptoms reported in this study were extremely minor.

The present study statistically analyzed the progress in clinical outcomes for 6 months. Although 6 months was not adequate to confirm postoperative side effects, only few patients returned for follow-up after 6 months. However, no notable changes were identified in patients who returned several years after the 6-month postoperative follow-up.

Some procedures used in this study require further research, while a few procedures are difficult to disclose. The associated complications need further research in the future.

For breast augmentation surgery using ADSCs and autologous fat, the amount of fat available for extraction from the patient’s body is limited. Moreover, even if a sufficient amount of fat is available for ADSCs, injection of excess fat is associated with a higher probability of complications, such as fat necrosis and calcification, making it challenging to achieve the desired breast size.

## CONCLUSIONS

Fat transplantation with ADSCs allows for quick recovery with minimum surgical scars and no immune response. The shape and natural feeling of the breasts are well maintained. Most patients are satisfied because foreign substances are not used. Despite consultation and explanation before surgery, some patients reported dissatisfaction with their breast size. Therefore, surgery should be delayed for patients who fail to completely understand and agree to the procedure. With standardized basic research and clinical studies, fat transplantation with ADSCs may become the gold standard approach for breast augmentation.

## Supplementary Material

ojaa007_suppl_Supplementary_Figure_1

ojaa007_suppl_Supplementary_Figure_2

ojaa007_suppl_Supplementary_Figure_3

ojaa007_suppl_Supplementary_Figure_4

ojaa007_suppl_Supplementary_Figure_5
