# Supplementary figures and images for: Breast Augmentation by Fat Transplantation With Adipose-Derived Stem/Stromal Cells
Source: Aesthet Surg J Open Forum. 2020 Feb 7;2(1):ojaa007. doi: 10.1093/asjof/ojaa007 (PMC7671271; doi:10.1093/asjof/ojaa007)

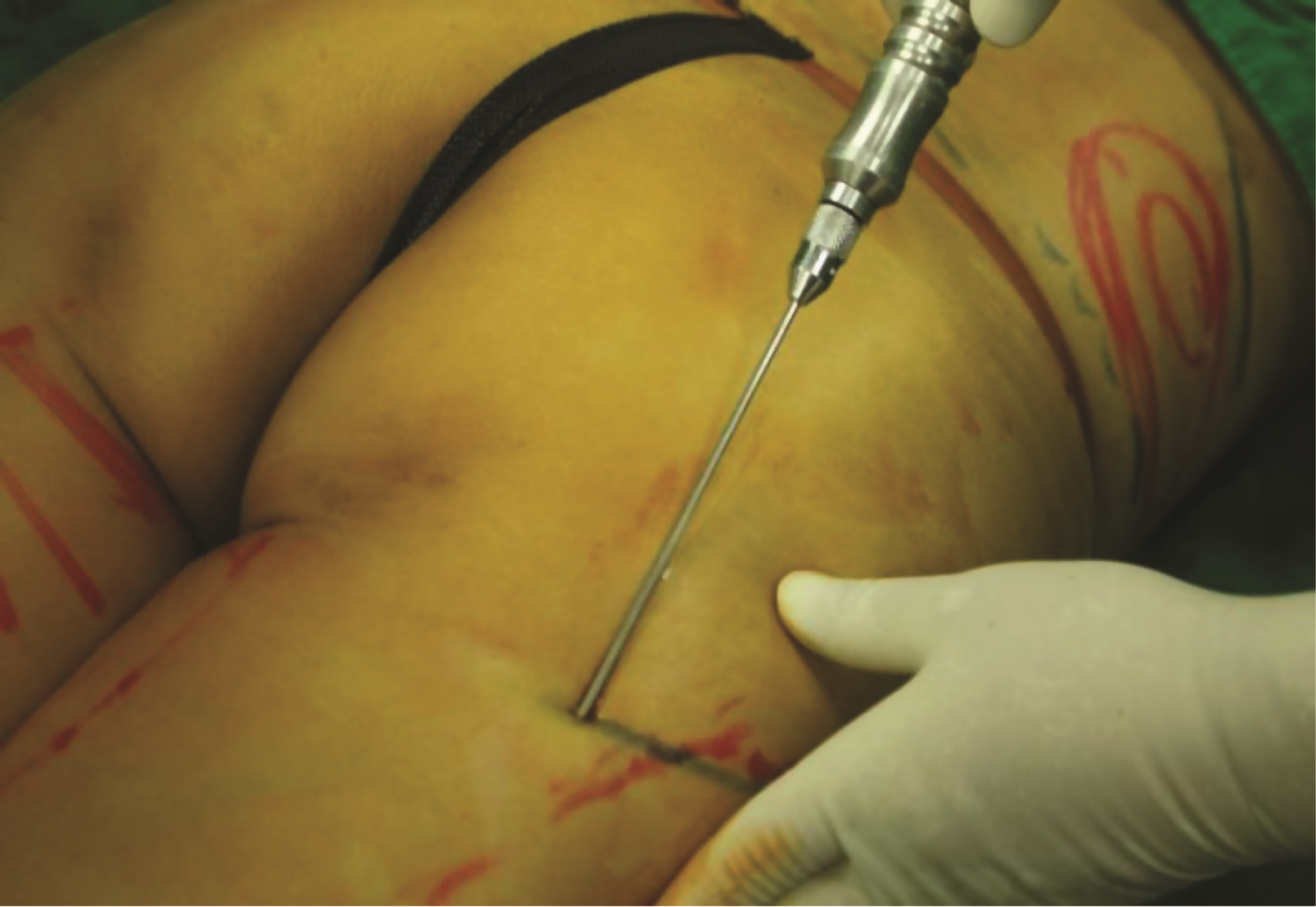

Supplement: ojaa007_suppl_Supplementary_Figure_1 [file ojaa007_suppl_supplementary_figure_1.png]

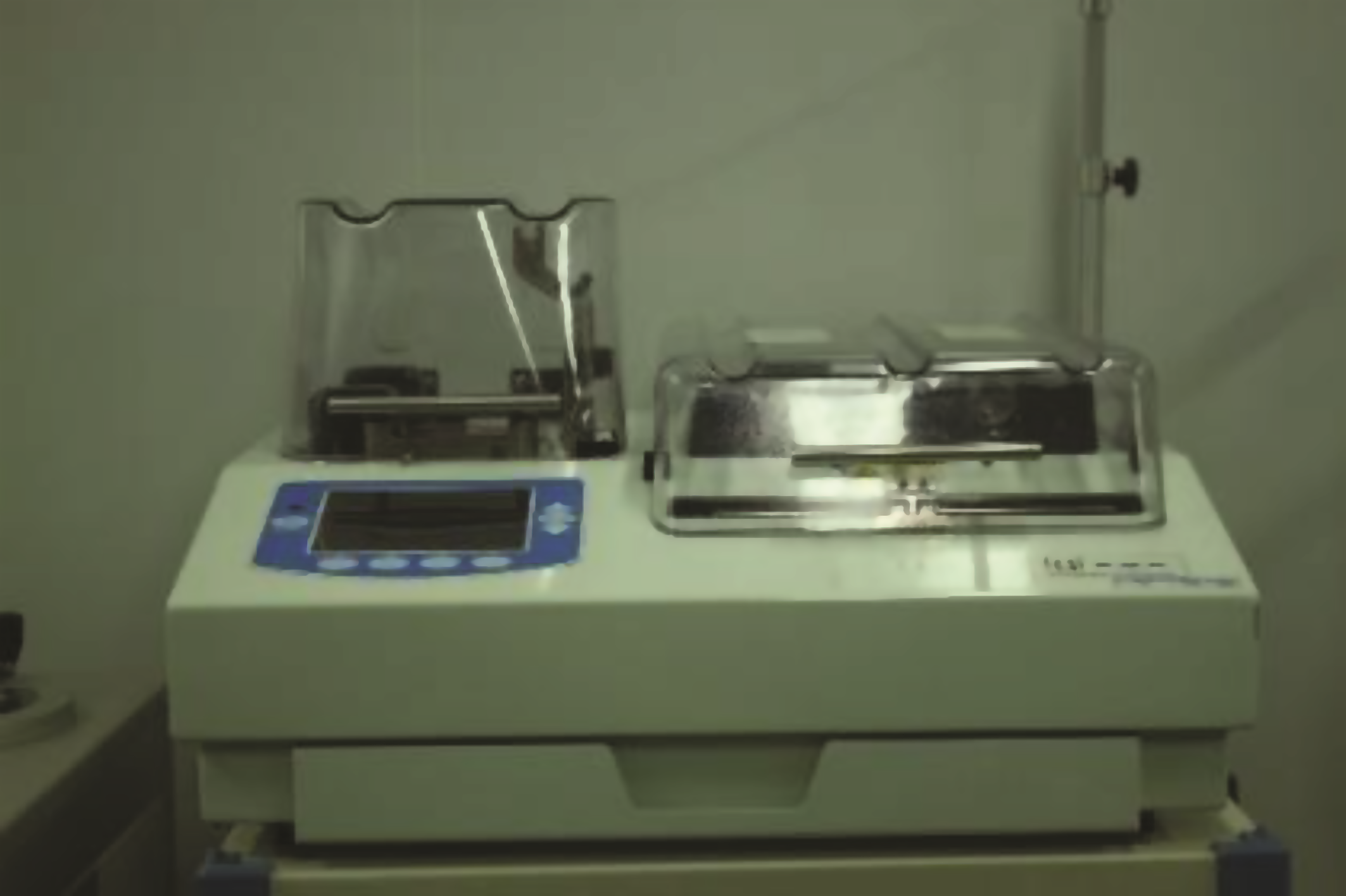

Supplement: ojaa007_suppl_Supplementary_Figure_2 [file ojaa007_suppl_supplementary_figure_2.png]

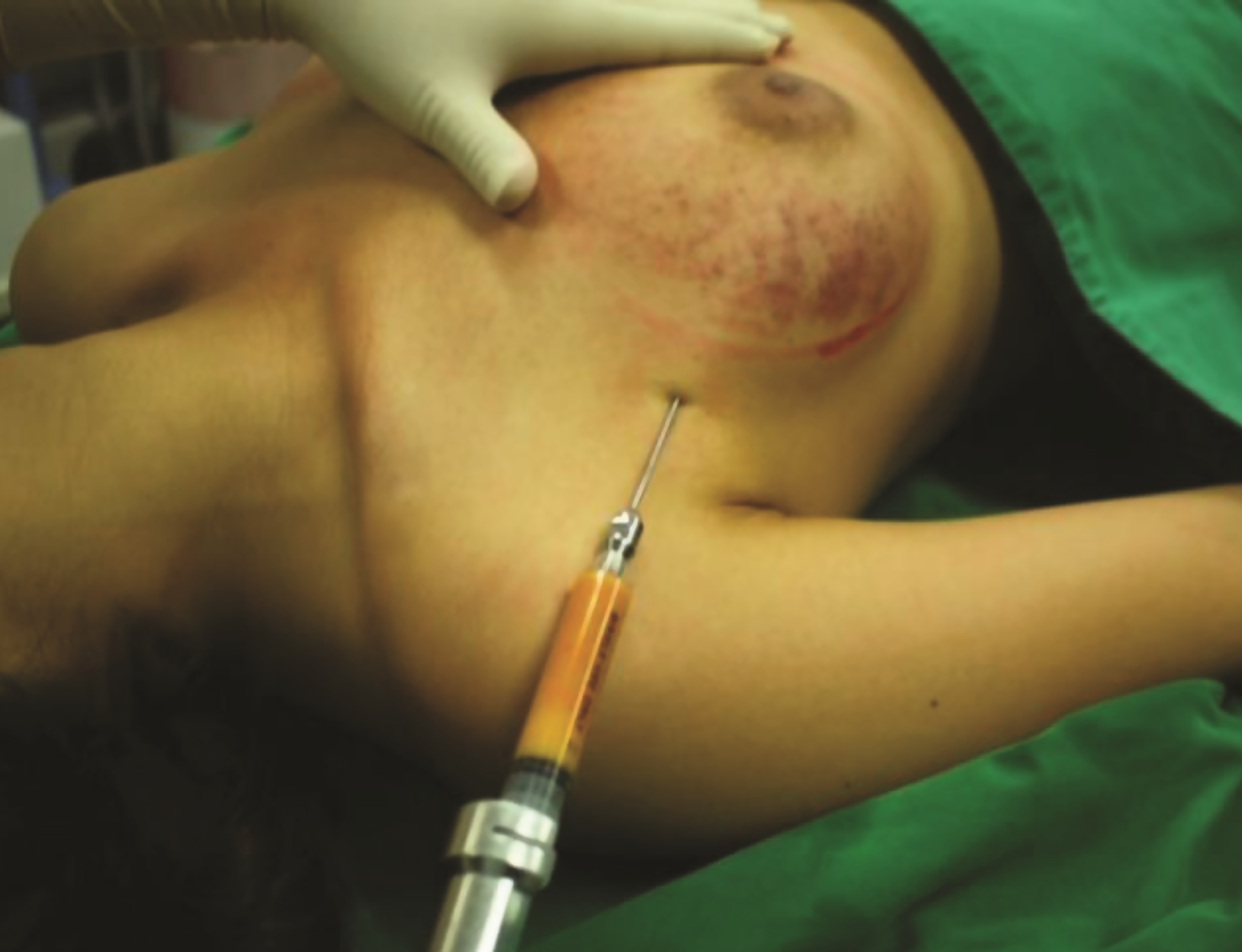

Supplement: ojaa007_suppl_Supplementary_Figure_3 [file ojaa007_suppl_supplementary_figure_3.png]

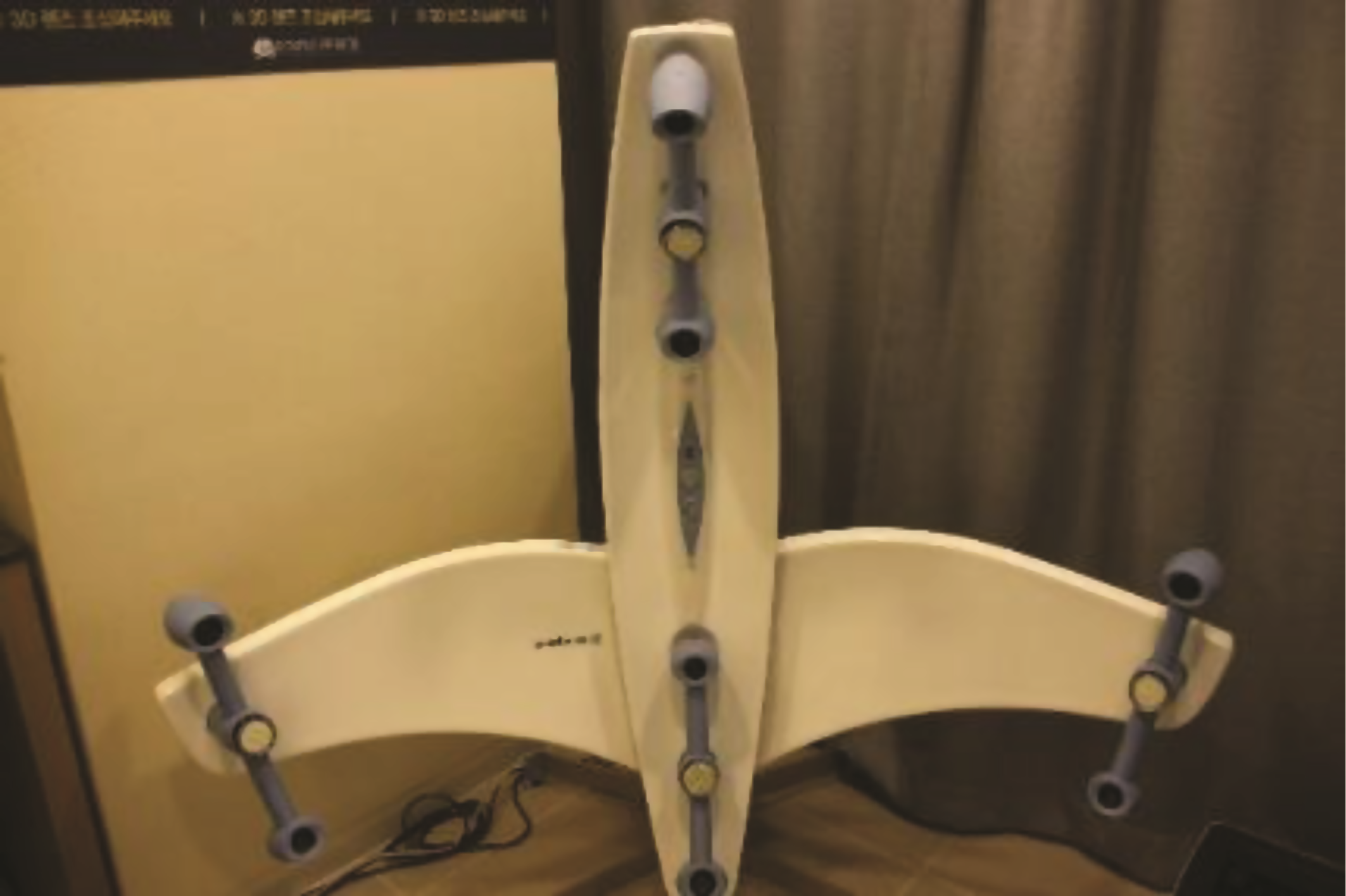

Supplement: ojaa007_suppl_Supplementary_Figure_4 [file ojaa007_suppl_supplementary_figure_4.png]

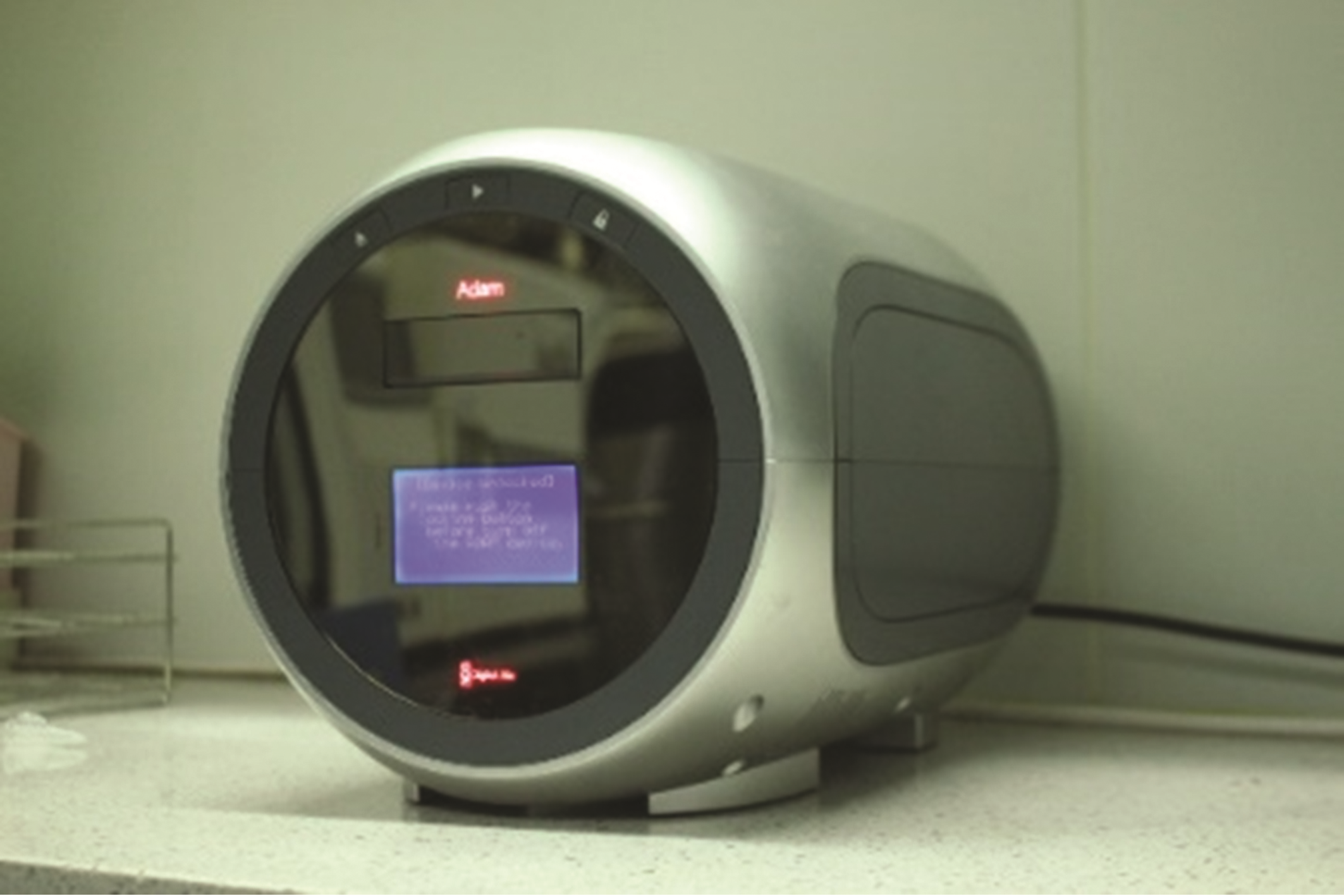

Supplement: ojaa007_suppl_Supplementary_Figure_5 [file ojaa007_suppl_supplementary_figure_5.png]
